# Genetic Spectrum Features and Diagnostic Performance of Four Plasma Biomarkers in 245 Patients with Frontotemporal Dementia

**DOI:** 10.1002/alz.087356

**Published:** 2025-01-09

**Authors:** Lu Shen, Tianyan Xu, Yafang Zhou, Cong Zhang, Beisha Tang, Bin Jiao, Ling Weng

**Affiliations:** ^1^ Xiangya Hospital, Central South University, Changsha, Hunan China

## Abstract

**Background:**

Frontotemporal dementia (FTD) exhibits clinical phenotypic and genetic heterogeneity. However, reports on the clinical phenotypic characteristics and the frequency of genetic mutations in large‐sample Chinese populations with FTD are lacking. Furthermore, the FTD diagnostic performance of plasma neurodegenerative biomarkers remains unclear.

**Method:**

This study included 245 clinically diagnosed FTD patients for an analysis of clinical characteristics. All FTD patients and 1,000 gender‐ and age‐matched healthy controls underwent targeted gene sequencing or whole‐genome sequencing. Additionally, plasma samples were analyzed for GFAP, α‐Syn, NfL, and P‐tau181 levels.

**Result:**

It was found that 44.5% (109/245) of patients were diagnosed with bvFTD, 21.2% (52/245) with SD, 15.5% (38/245) with PNFA (20/245), 10.6% (26/245) FTD‐PD, and 8.2% with FTD‐ALS. After gene sequencing, 53 pathogenic or likely pathogenic (P/LP) variants in FTD‐related genes were identified in a total of 19.2% of the patients (47/245). The most common mutation was MAPT (4.9%, 12/245), followed by C9orf72 (3.3%, 8/245), CHCHD10 (2.4%, 6/245), and GRN (1.6%, 4/245) mutations. Other rare variants together were found in the other eleven genes. Elevated levels of GFAP, α‐Syn, NfL, and P‐tau181 were significantly observed in FTD patients compared to controls (P < 0.05), and among them, a combination of GFAP, α‐syn, and Nfl demonstrated high diagnostic value for varied FTD subtypes, with AUCs of 0.83‐0.93. We further confirmed that the gene variants could not have an impact on plasma biomarkers level.

**Conclusion:**

This research enhances comprehension regarding the genetic and phenotypic spectrum of FTD within the Chinese population, and underscores the diagnostic utility of biomarkers such as GFAP, α‐Syn, and NfL in identifying FTD.

